# Prediction for Origin-Destination Distribution of Dockless Shared Bicycles: A Case Study in Nanjing City

**DOI:** 10.3389/fpubh.2022.849766

**Published:** 2022-04-08

**Authors:** Min Cao, Ying Liang, Yanhui Zhu, Guonian Lü, Zaiyang Ma

**Affiliations:** ^1^Key Laboratory of Virtual Geographic Environment (Ministry of Education of PRC), Nanjing Normal University, Nanjing, China; ^2^State Key Laboratory Cultivation Base of Geographical Environment Evolution (Jiangsu Province), Nanjing Normal University, Nanjing, China; ^3^Jiangsu Center for Collaborative Innovation in Geographical Information Resource Development and Application, Nanjing Normal University, Nanjing, China

**Keywords:** dockless shared bicycles, origin and destination (OD), OD distribution, QPSO, LSTM

## Abstract

Shared bicycles are currently widely welcomed by the public due to their flexibility and convenience; they also help reduce chemical emissions and improve public health by encouraging people to engage in physical activities. However, during their development process, the imbalance between the supply and demand of shared bicycles has restricted the public's willingness to use them. Thus, it is necessary to forecast the demand for shared bicycles in different urban regions. This article presents a prediction model called QPSO-LSTM for the origin and destination (OD) distribution of shared bicycles by combining long short-term memory (LSTM) and quantum particle swarm optimization (QPSO). LSTM is a special type of recurrent neural network (RNN) that solves the long-term dependence problem existing in the general RNN, and is suitable for processing and predicting important events with very long intervals and delays in time series. QPSO is an important swarm intelligence algorithm that solves the optimization problem by simulating the process of birds searching for food. In the QPSO-LSTM model, LSTM is applied to predict the OD numbers. QPSO is used to optimize the LSTM for a problem involving a large number of hyperparameters, and the optimal combination of hyperparameters is quickly determined. Taking Nanjing as an example, the prediction model is applied to two typical areas, and the number of bicycles needed per hour in a future day is predicted. QPSO-LSTM can effectively learn the cycle regularity of the change in bicycle OD quantity. Finally, the QPSO-LSTM model is compared with the autoregressive integrated moving average model (ARIMA), back propagation (BP), and recurrent neural networks (RNNs). This shows that the QPSO-LSTM prediction result is more accurate.

## Introduction

Shared bicycle systems have been widely adopted in cities as a promising solution to the last mile issues in public transportation (1–3). Apart from the immediate advantage for city commuters, it can also provide benefits to the environment and public health (4–8). In particular, due to its advantages of reducing resource consumption and chemical emissions, the use of shared bicycles is more environmentally friendly than motorized transportation (9). There is little doubt that using shared bicycles rather than automobiles benefits the environment. Meanwhile, the application of shared bicycles has the potential to promote public health. Physical inactivity has been linked to increased morbidity and mortality in numerous studies (10). Shared bicycles can provide means for people to exercise and improve their overall health (5, 11, 12).

At present, there are two types of shared bicycles in Nanjing city, docked shared bicycles and dockless shared bicycles. The docked shared bicycles require unified management with hardware devices, such as parking piles and paying devices. However, the docked shared bicycle user experience is not good due to the cumbersome certification and registration process (13). Moreover, the high capital and space costs of its supporting equipment also limit its development to a certain extent. Based on the mobile internet technology, dockless shared bicycles have rapidly developed in China and have become the main means of shared bicycles in the market due to their better user experience, simple registration and certification process, and convenience afforded when borrowing and returning bikes (14, 15). Dockless shared bicycles provide residents with more convenient services due to their stop-on-ride, flexibility, ease of use and low price. However, there are also many factors that are not conducive to the development of urban transportation, such as disorderly parking and the imbalance between supply and demand (16–18). These problems might decrease the opportunity and willingness of the public to use shared bicycles, but they have occurred in almost every city where shared bicycles are deployed in Asia, Europe and the Americas, including Beijing, Shanghai and Nanjing in China (17, 19, 20).

Short-term forecasts for the origin and destination (OD) number of shared bicycles in different areas can help in the discovery of behavioral patterns and help to solve the above possible future problems in advance (21). With better prediction results, users can be informed of the distribution of bikes in an area sometime in the future to better plan their itinerary. Operators can plan to place and reallocate bikes to improve the customer experience according to short-term demand forecasts. This will further promote the travel efficiency of urban residents and accelerate the improvement of the city's green travel layout. Therefore, it is beneficial not only for users and operators but also for the environment and public health. Geographic modeling is a useful way to discover geographic patterns and predict geographic processes (22, 23). Many scholars have utilized different models for shared bicycle prediction, including linear models based on mathematical statistics, intelligent theoretical models represented by neural networks, and combined models, which combine more than two types of models. The more representative linear model is the autoregressive integrated moving average (ARIMA) model. The ARIMA model was applied to predict the flow number and trip duration of bicycles (24). Although ARIMA is the most common statistical model for time series prediction, it has extremely high data requirements and requires time series data to be stable. It only captures the linear relationship of the data. The intelligent theoretical models used for bike-sharing prediction mainly include back propagation (BP), recurrent neural networks (RNNs), and long short-term memory (LSTM). BP has been used to predict shared bicycle demand and the number of public bicycle rented (25, 26). However, its learning speed is very slow, and network training is more likely to fail. RNNs are particularly good at capturing the temporal and spatial evolution of traffic flow, quantity and speed, so they are often used to predict short-term traffic volumes (27, 28). Although traditional RNNs perform well in non-linear time series data modeling, there are still several issues to be addressed, such as the inability to train time series with long time lags and the difficulty of automatically finding the optimal time window size (29, 30). LSTM makes up for the gradient disappearance and gradient explosion of RNNs and the lack of long-term memory ability so that the recurrent neural network can make full use of the long-term sequence information (31). Thus, LSTM can be applied to predict traffic flow and the demand of dockless shared bicycles (32). Due to the complexity of the traffic system and the shortcomings of various models, scholars have combined multiple models in recent studies to make full use of the advantages of different models for shared bicycle prediction. Combined models usually combine several different intelligent theoretical models and are based on mathematical statistics and intelligent theoretical models. For example, convolutional neural networks (CNNs) and LSTM were combined to predict the short-term distribution of dockless shared bicycles (33); a combination of CNNs and LSTM in a deep learning model was applied to predict the travel distance and OD distribution of shared bicycles (34).

The smart prediction of shared bicycles in this article is based on a deep learning algorithm. The first condition for accurate analysis using machine learning is to determine the appropriate model structure, including the number of stacked layers, the number of layer nodes, the activation function, the batch size and other hyperparameters. Determining the optimal hyperparameter combination in a high-dimensional space is a complicated problem. The traditional method for determining hyperparameters is the manual parameter adjustment method, which mainly relies on the experience of researchers, has strong subjectivity, and requires a long time for conducting experiments. In addition, other hyperparameter optimization methods, such as grid search, random search and Bayesian optimization, have high time complexity. There have been many studies on optimizing hyperparameters by using swarm intelligence algorithms due to their excellent parameter optimization performance. Commonly used swarm intelligence algorithms include ant colony optimization (ACO), particle swarm optimization (PSO), and quantum particle swarm optimization (QPSO) (35, 36). They can be applied to various types of deep learning hyperparameter optimization, but they were mainly applied to optimize common models such as support vector machines (SVMs) and BP (37–42).

Most short-term demand predictions were based on regular grids. Few studies use swarm intelligence algorithms to optimize models with more parameters, such as LSTM, and even fewer use them for shared bicycle prediction. This article aims to build a comprehensive model, called QPSO-LSTM, for predicting the OD quantity of dockless shared bicycles by combining QPSO and LSTM models. By taking AOI (area of interest) as the basic analysis unit, the model attempts to consider the influence of different urban function types on bicycle distribution. In addition, QPSO is applied to optimize LSTM to obtain better predictions. By taking the main urban areas of Nanjing city as an example, the QPSO-LSTM model is applied to predict the OD number of dockless shared bicycles in different types of AOIs. It can help determine possible problems in advance, such as bike aggregation or an insufficient number of bikes in the future period, to help with the bike scheduling and promote bike distribution rebalancing. Finally, the model is compared with other commonly used models, including BP, ARIMA and RNN, to verify its accuracy.

## Study Area and Data Processing

### Study Area and Data Acquisition

**Figure 1:**
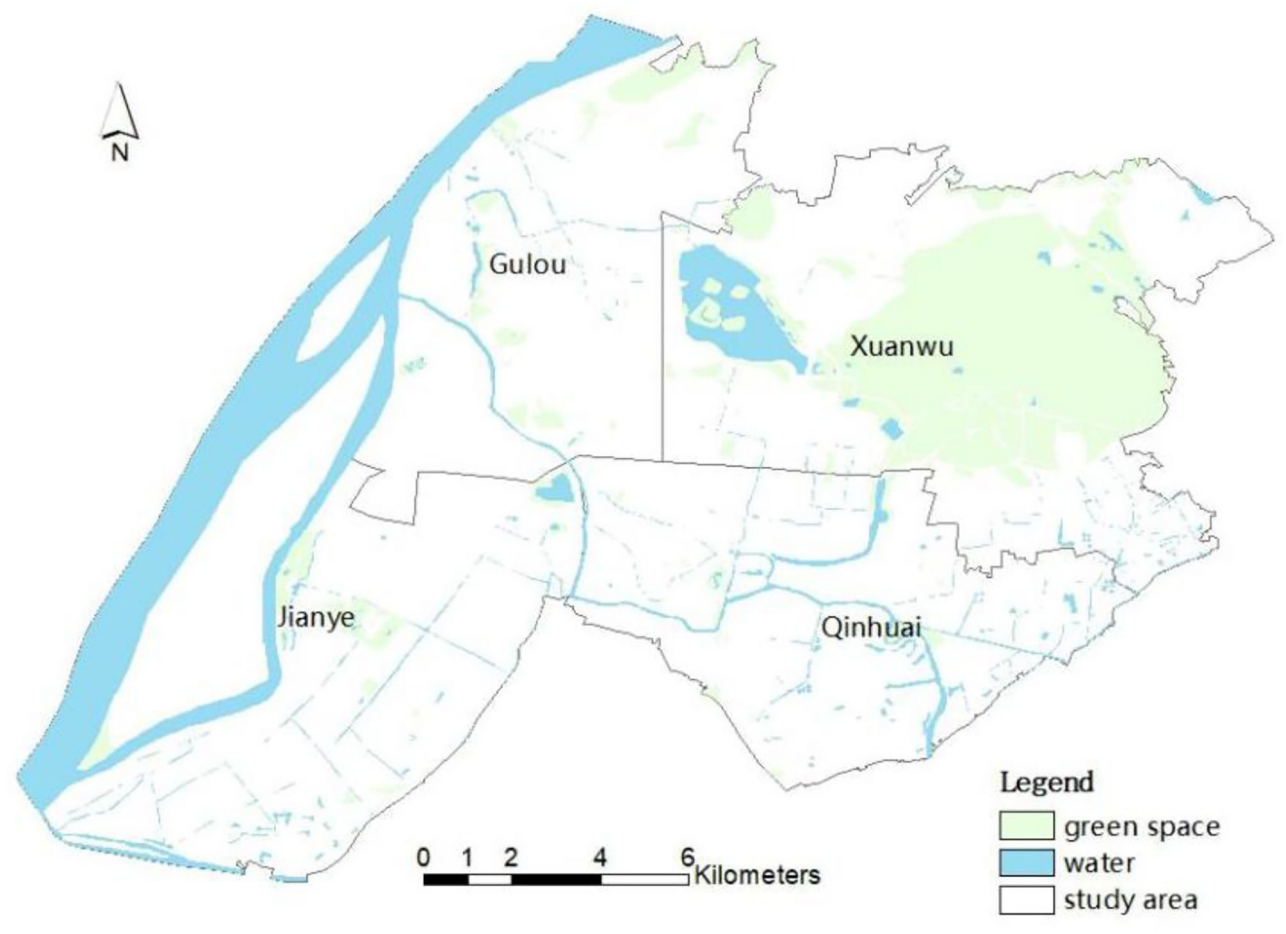
Study area.

Nanjing is an important gateway city for the development of the Yangtze River Delta. According to the 7th census, it has a permanent population of ~9 million (2021). Since 2015, shared bicycles have experienced rapid development in Nanjing and have become a new travel choice for residents. Dockless shared bicycles were born as a new form of the sharing economy at the end of 2016, including bike share systems such as Ofo, Mobike and Hello Bike. Dockless shared bicycles quickly swept across the country due to their higher flexibility. As reported by Jiangsu Sina (2019), in 2018 the number of shared bicycles in Nanjing reached a record high of ~600,000–700,000. Then the total number of shared bicycles was limited to ~317,000 to regulate the bike-sharing market under the control of the government. According to the Hello Bike use report, in Nanjing (2017), 40,000 shared bicycles have been used 31 million times in 3 months, covering a distance of 54.81 million km, equivalent to cycling 1,367 laps around the equator. In this article, the study area is the downtown area of Nanjing, including Xuanwu, Gulou, Jianye and Qinhuai Districts, as shown in Figure 1.

Bicycle share ridership is affected by different weather variables, socio-demographic attributes, land use and built environment, among which the impact of several weather variables on bicycle trips has been investigated in many studies (43–49). This article obtains the datasets of dockless shared bicycles, road networks, traffic facility points, AOI data and administrative zoning data for Nanjing, as shown in Table 1. The dockless shared bicycle dataset includes the bike location data, including the date, bike ID, longitude and latitude of each bike from March 9 to April 8, 2018. The location data of 105,901 shared bicycles in the study area over 32 days include ~100 million records. The meteorological data, including temperature, wind speed, and precipitation from March 8 to April 2, 2018, with an interval of 1 h, were added as factors affecting bicycle trips. AOI is the area data used to represent each geographical entity, such as separate residential areas, independent commercial areas, and scenic zones. It is a carrier of all the social and economic activities of residents and reflects different types of urban functions. The attributes of each AOI include the name, address, category and latitude and longitude. The AOI is taken as the basic analysis unit in the study.

**Table 1:** Data acquisition in the study area.

**Types of data**	**Data description**
Dockless shared bicycle dataset	Bike location data obtained from the mobile clients of Ofo and Mobike every 15 min
AOI data	Gaode map AOI data (data source: https://lbs.amap.com/)
Urban administrative zoning data	Shapefile of urban administrative zoning (data source: http://www.tianditu.gov.cn/)
Meteorological data	NCDC (National Climatic Data Center, China)

### Periodicity of Shared Bicycle Trips

#### Time Interval of Periodic Series

**Figure 2:**
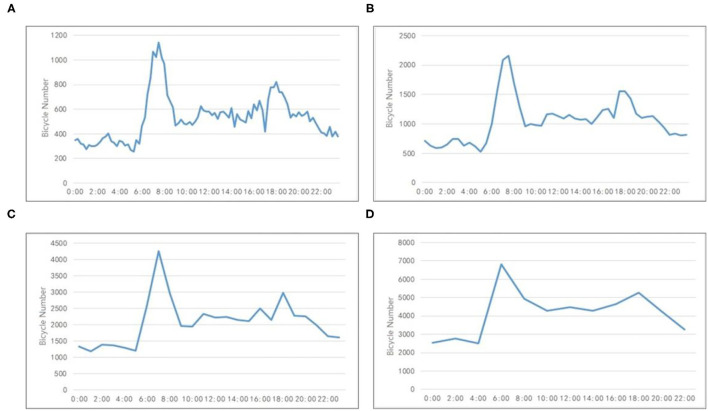
The curves of bicycle numbers at different intervals. **(A)** 15 min. **(B)** 30 min. **(C)** 60 min. **(D)** 120 min.

The time interval for shared bicycle location data is 15 min. The number of bicycles in different time intervals was analyzed to determine the appropriate time interval for our study. Figure 2 shows the curves of bicycle numbers at 15, 30, 60, and 120 min intervals at the original points of residential areas of the study area on March 14. Figure 2 shows that the curve of the 1-h time interval can express the regularity of bicycle changes, and the curve is smoother than the time interval of 15 and 30 min. When the interval is >60 min, there are some difficulties in discovering the number changes of bicycle trips in 1 day. Therefore, this article takes 1 h as the time interval during the experiment to alleviate the impact of possible data loss on the overall law and effectively reduce the calculation rate.

#### Similarities in Different Types of AOIs

The possible trend similarity is explored to reflect the similar trends of cycling in different types of AOIs in the same time period. The cosine similarity can measure the trend similarity by comparing two vectors in a vector space. It evaluates the similarity between two vectors by calculating the cosine of the angle between them; thus it is not related to the specific value and is only affected by the angle of the two vectors. Formula (1) is used to calculate the cosine similarity cosθ (50).


(1) $$\cos\theta =\frac{\sum_{i=1}^{n}A_{i}B_{i}}{\sqrt{\sum_{i=1}^{n}{\left(A_{i}\right)}^{2}}\sqrt{\sum_{i=1}^{n}{\left(B_{i}\right)}^{2}}}$$

where cosθ is the cosine similarity; *A*_*i*_ and *B*_*i*_ represent the components of vectors *A* and *B*, respectively; and *n* is the number of samples.

Table 2 lists the calculated cosine similarities among the AOIs of residential areas, commercial buildings and scenic areas. A higher cosine similarity indicates that the variation trend in the number of bicycle trips is similar within the same type of AOI. In contrast, a lower cosine similarity indicates that the variation trend in the number of bicycle trips is different in various types of AOIs, which are located in different urban function regions.

**Table 2:** Trend similarity in different types of AOIs.

**Cosine similarity**	**Residential area**	**Commercial building**	**Scenic area**
Residential area	0.762	0.529	0.584
Commercial buildings		0.737	0.517
Scenic area			0.687

#### Daily Correlation

The daily changes in the number of bicycle trips on weekdays and weekends are considered to be different. The Pearson correlation coefficient is applied to calculate the daily correlation of the numbers of bicycle rides every 2 days in a week from March 12 to March 18. Formula (2) can calculate the Pearson correlation coefficient ρ_*X, Y*_ (51).


(2) $$\rho_{X,Y}=\frac{\sum_{i=1}^{n}\left(X_{i}-\bar{X})(Y_{i}-\bar{Y}\right)}{\sqrt{\sum_{i=1}^{n}{\left(X-\bar{X}\right)}^{2}}\sqrt{\sum_{i=1}^{n}{\left(Y-\bar{Y}\right)}^{2}}}$$

where *X, Y* are 2 days; *n* is the number of hours in each day, which is 24; *X*_*i*_, *Y*_*i*_ are the number of bicycles at the *i*th hour corresponding to *X, Y*; and $\bar{X},\bar{Y}$ are the average values of *X, Y*. When ρ_*X, Y*_ is −1, it means that *X, Y* are completely negatively correlated; when ρ_*X, Y*_ is 1, it means that *X, Y* are completely positively correlated; when ρ_*X, Y*_ is 0, it means that *X, Y* have no correlation.

As shown in Table 3, the correlation coefficients between weekdays are higher than 0.9. However, the correlation coefficients between weekdays and weekends are lower, ranging from 0.8 to 0.9. The correlation coefficient between Saturday and Sunday is 0.973, showing a high correlation. The results show that the daily cycling regularity is similar among weekdays and between weekends, but it is slightly different between weekends and weekdays.

**Table 3:** Daily correlation in 1 week.

**Similarity**	**Monday**	**Tuesday**	**Wednesday**	**Thursday**	**Friday**	**Saturday**	**Sunday**
Monday	1	0.970	0.965	0.957	0.974	0.848	0.788
Tuesday		1	0.940	0.918	0.957	0.811	0.723
Wednesday			1	0.954	0.970	0.819	0.759
Thursday				1	0.971	0.878	0.849
Friday					1	0.879	0.824
Saturday						1	0.973
Sunday							1

Above all, the time interval for shared bicycle location data is determined to be 1 h in the constructed QPSO-LSTM model. In addition, due to the different regularity of changes, the number of bikes on weekdays and that on weekends need to be predicted separately, as does that in different AOIs.

## Model Construction

**Figure 3:**
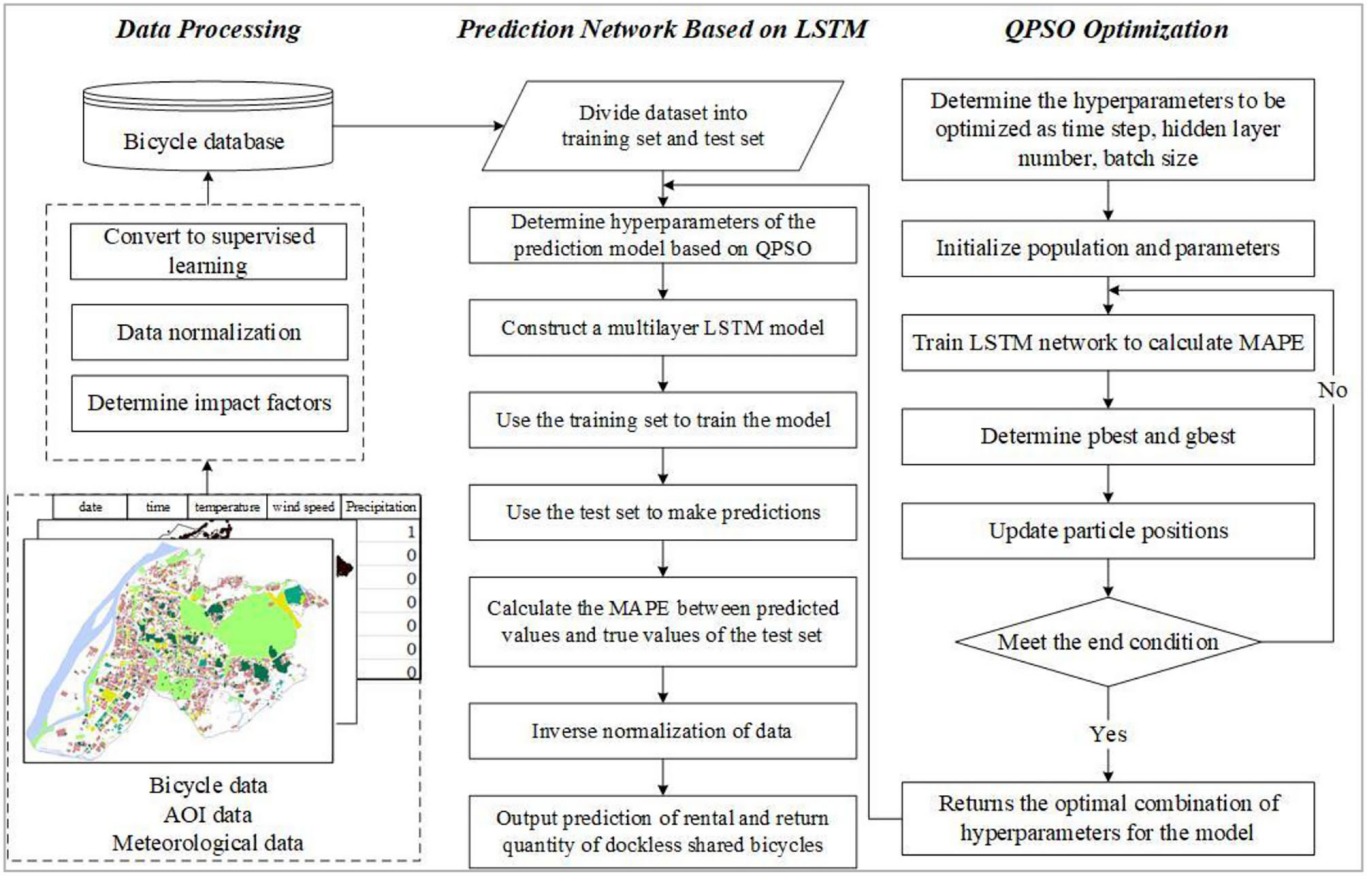
QPSO-LSTM flowchart.

The QPSO-LSTM model for predicting the OD quantity of shared bicycles is constructed as shown in Figure 3. The model is trained by using a training set and then is used to predict the number of shared bicycle trips in the future by using a test set. The model implementation process consists of three parts: data processing, the prediction model based on LSTM, and QPSO optimization.

First, the acquired cycling data were classified according to different AOI types to form cycling data with an interval of 1 h. Temperature, wind speed and precipitation were selected as the influencing factors of cycling and incorporated into the cycling data. The data underwent normalization, supervised learning and dataset division processing before model training and prediction. In particularly, 80% of the data were used as the training set and 20% were used as the validation set (52). Then, as a deep learning algorithm for sequential data prediction, LSTM is the core algorithm of the prediction model QPSO-LSTM. It was used to predict the OD quantity of shared bicycles by building a multilayer LSTM network. Finally, QPSO was applied to solve the optimization problem of the hyperparameters of the prediction model. By using QPSO, the hyperparameter combination suitable for the prediction model can be determined quickly to effectively improve the accuracy of the model.

### Prediction Network Based on LSTM

**Figure 4:**
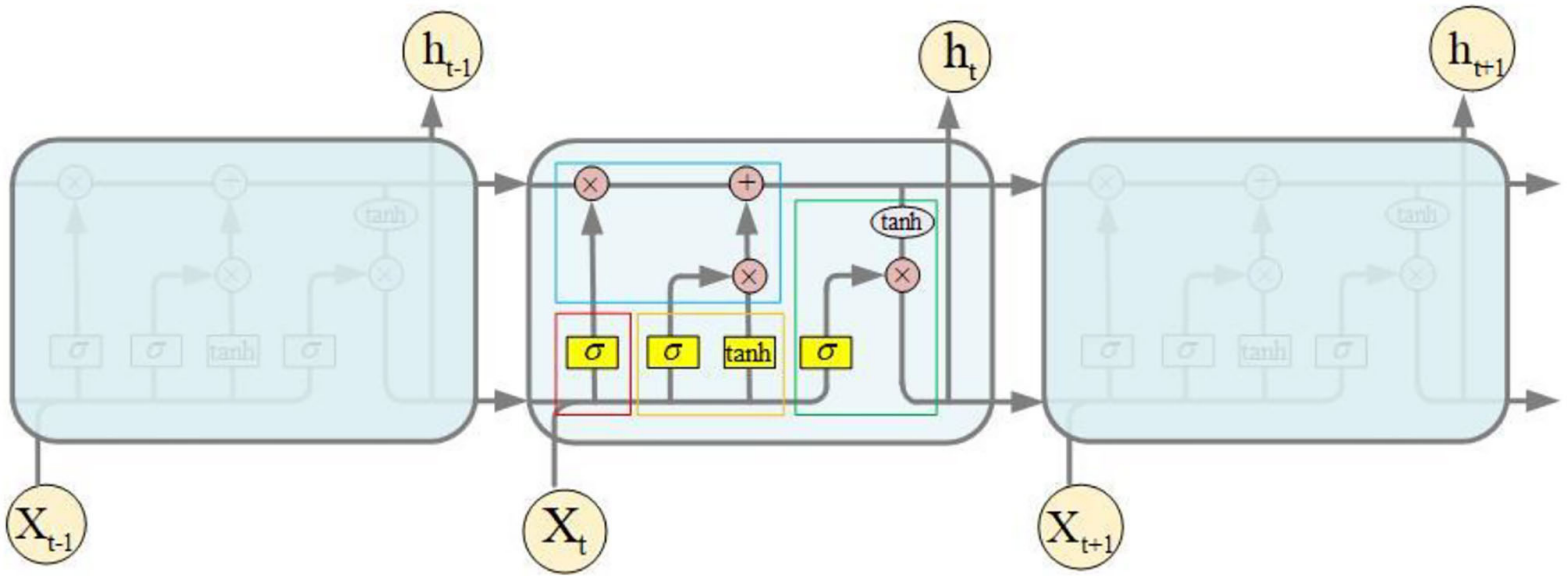
Prediction network of QPSO-LSTM: Redrawn and extended from Li et al. (53).

The structure of the LSTM network of QPSO-LSTM is shown in Figure 4 below. Its input data are bicycle and meteorological data, and the output data are the number of bicycles needed in the future. The horizontal line through the entire memory cell is similar to a conveyor belt, indicating the state of the cell. There are three types of gates in LSTM: the forget gate, input gate and output gate. The forget gate determines how much information should be removed from the cell state at the last moment, as shown in the red box in Figure 4. The new input information is determined by the input gate in the cell state, as shown in the yellow box. The parts of the cell state that are utilized to generate the final output of the memory cell are decided by the output gate, as shown in the green box (54–56). In addition, the pink circle in the memory cell represents the point operation, while the yellow rectangle represents the activation function, and they are all used to compute the gates and the memory cell output.

### QPSO Optimization

QPSO hyperparameter optimization mainly includes determining the parameters to be optimized, selecting the fitness function, and determining and updating the optimal positions of particles.

**Figure 5:**
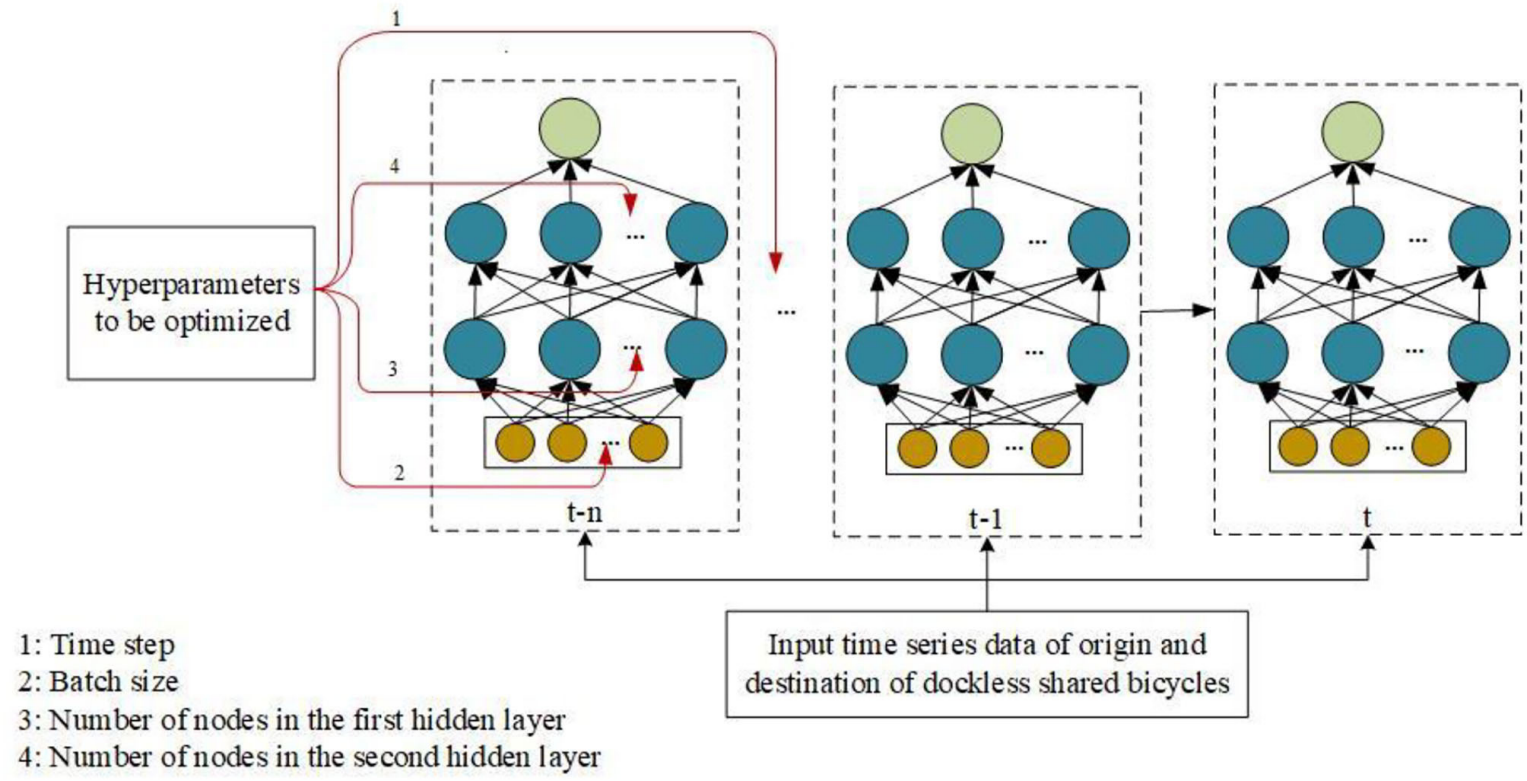
Parameters to be optimized in the prediction network: 4 parameters are selected to be optimized.

As shown in Figure 5, the time step, batch size and the numbers of nodes in the network hidden layers are selected as the parameters to be optimized in the prediction network, and each has an arrow pointing to it. The time step parameter determines the number of previous data moments in the model input at a certain moment. The batch size can effectively improve memory utilization and model training speed after optimization. A reasonable number of hidden layer nodes can avoid as much of the model over fitting phenomenon during training as possible. The change in the number of shared bicycle trips has a daily characteristic, so the value range of the time step is determined to be [1, 24]. The number of hidden layer nodes is determined according to the empirical rule (25), as shown in Formula (3).


(3) $$\begin{array}{l} u=\sqrt{m+n}+a \end{array}$$

where *m, n* are the number of input and output layers of the network, respectively, and *a* is any integer between 3 and 10. The number of hidden layers ranges from [5, 50], and the range of the batch size is set to [1, 30].

QPSO needs to determine the objective function to determine the pros and cons of the current particle. Commonly used evaluation indicators for regression models include root mean square error (RMSE), mean absolute error (MAE) and mean absolute percentage error (MAPE) (57). Formulas (4–6) below are used to calculate these evaluation indicators.


(4) $$\begin{array}{l} RMSE\;=\;\sqrt{\frac{1}{m}\sum_{i=1}^{m}{\left[h\left(x_{i}\right)-y_{i}\right]}^{2}} \end{array}$$

(5) $$\begin{array}{l} MAE\;=\;\frac{1}{m}\overset{m}{\underset{i=1}{\sum }}|h\left(x_{i}\right)-y_{i}| \end{array}$$

where *m, h*(*x*_*i*_), and *y*_*i*_ are the length, predicted value and true value of the verification set, respectively.

When using QPSO to optimize the hyperparameters of our prediction network, MAPE is selected as the fitness function of the particle, which is defined by Formula (6).


(6) $$MAPE=\frac{1}{m}\sum_{i=1}^{m}\left|\frac{h\left(x_{i}\right)-y_{i}}{y_{i}}\right|$$

According to the fitness function, the objective function value, the MAPE between the predicted value and the true value of the model, at each position is calculated. The smaller the objective function value is, the better the position.

In the whole learning process, the optimal position found by individual particles is *pbest*_*i*_, and the mean value of the best position of individual particles is *mbest*. The objective function values of all particles in the population are calculated many times during the learning process to determine the global optimal position *gbest* of each particle in all the learning processes. Formulas (7–9) can be used to calculate these values (35).


(7) $$\begin{array}{l} x_{i}\;=\;P_{i}\pm \alpha |mbest-x_{i}|\ln\left(\frac{1}{u}\right) \end{array}$$

(8) $$\begin{array}{l} P_{i}\;=\;\emptyset pbest_{i}+\left(1-\emptyset \right)gbest \end{array}$$

(9) $$\begin{array}{l} mbest\;=\;\frac{1}{M}\overset{M}{\underset{i-1}{\sum }}pbest_{i} \end{array}$$

where *M* is the size of the particle group; *x*_*i*_ is the position of the *i*th particle; α is the innovation parameter; and ∅ and *u* are uniformly distributed values on (0, 1).

## Model Application and Verification

### Model Results and Typical Case Analysis

The prediction models of different AOIs obtained after model training can be applied to predict the OD quantity of bicycles for all types of AOIs in the whole study area. According to the QPSO optimization results, the time step is 4, which means that the model uses the number of bicycles in the previous 4 moments to predict the number of bicycles in the next moment. In addition, the batch size and the node numbers of the first hidden layer and the second hidden layer are set to 28, 10, and 16, respectively.

**Figure 6:**
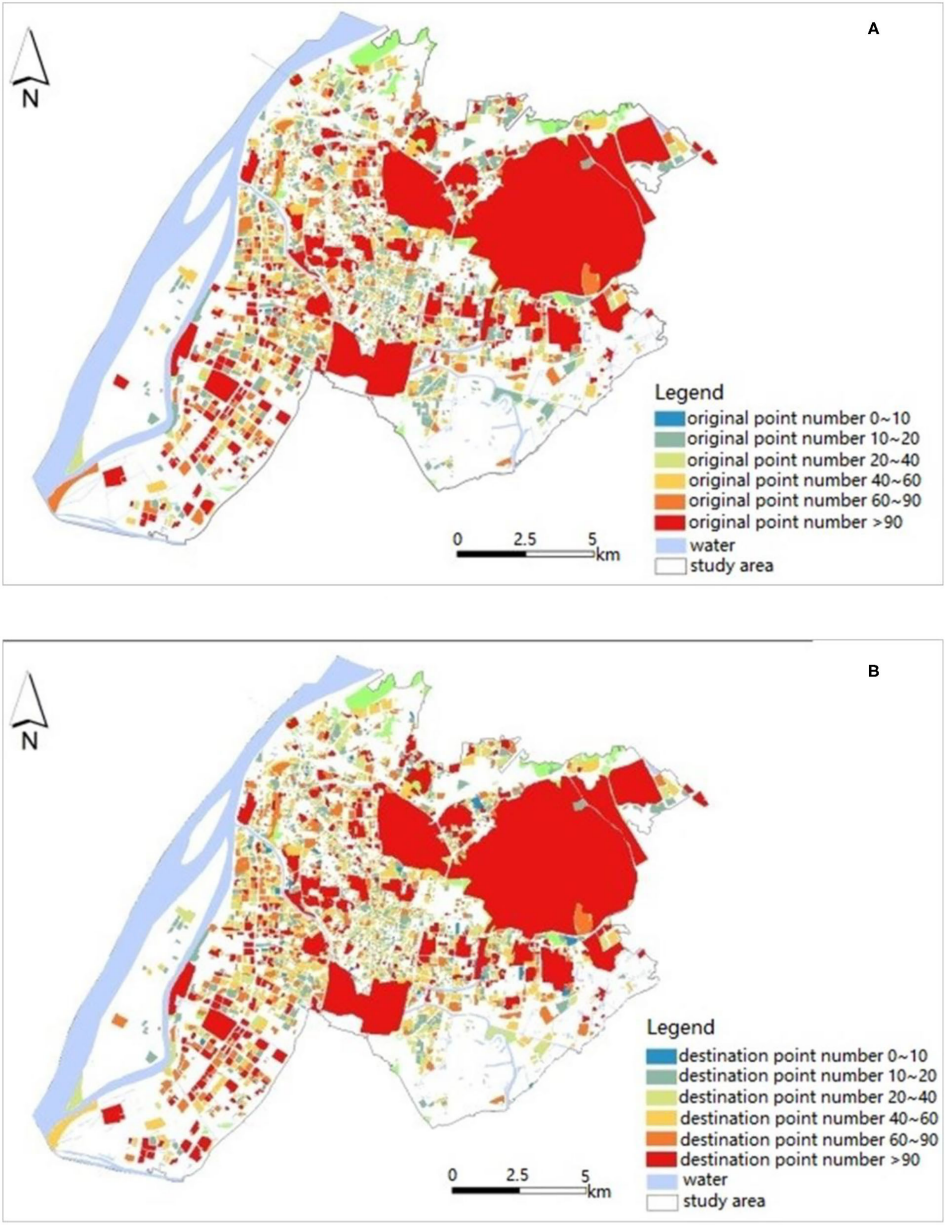
Prediction results at 8 am of study area. **(A)** Origin points. **(B)** Destination points.

For each AOI, the model to which it belongs is applied to predict the number of bicycles in each hour. For example, the prediction results of the OD quantities of bicycle rides at 8 am on March 14 are shown in Figure 6. At 8 A.M. in the study area, the bicycle OD quantities of the different regions are slightly different, which fully reflects the characteristics of residents' trips and verifies the effectiveness of the prediction model. As shown in the Figure 6, there are more origin points than destination points in residential areas. The AOI area of scenic areas accounts for the largest proportion of the total area of the study area, ~40.2%. Due to their larger area, there are more bikes in scenic areas, such as Purple Mountain and Xuanwu Lake, which are generally shown in red in the Figure 6.

**Figure 7:**
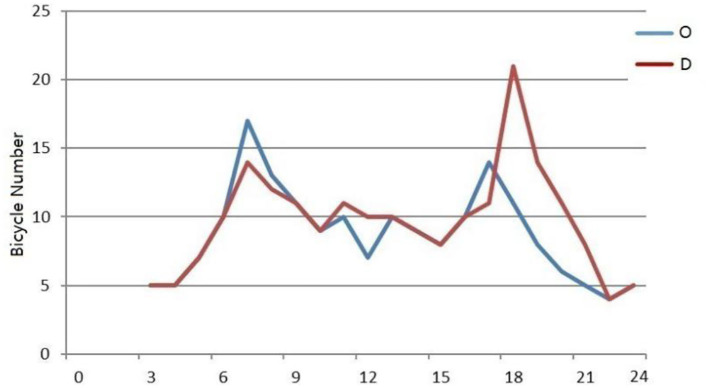
Prediction of bicycle OD quantity in Huaxincheng.

Two typical areas, including residential areas and commercial buildings, are selected to use the corresponding model to predict the OD quantity of bicycles. The typical residential area selected is Huaxincheng, which is located near Yuantong station of subway Line 1 and Line 2, and leisure and entertainment facilities such as Hexi Central Park and Nanjing Famous Taiwan Goods City are around it. Taking it as an example, its bicycle OD data on the weekday of March 14 are selected to predict the supply and demand of bicycles by using the prediction model of residential area AOIs. The prediction of bicycle OD numbers in Huaxincheng is shown in Figure 7. The results show that bicycle OD numbers are higher in the morning and evening peak hours, but their changes are different. The peak value of the number of origin points in the morning peak period is greater than that in the evening peak period, while the peak value of the destination points in the evening peak period is higher than that in the morning peak period. This prediction conforms to the daily regularity of cycling for residents commuting to work.

**Figure 8:**
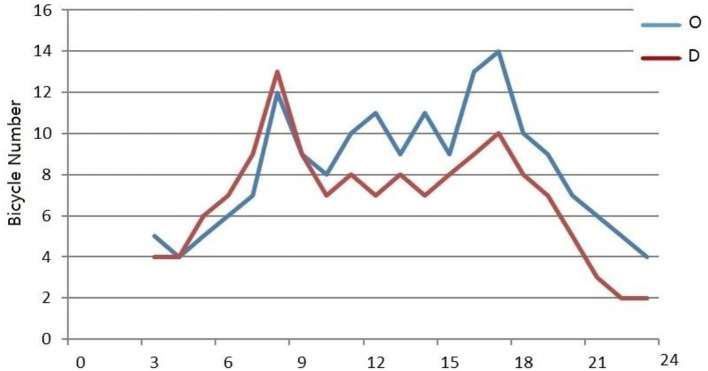
Prediction of bicycle OD quantity in Fenghuang Square.

The typical commercial building selected is Fenghuang Square located at No. 1, Hunan Road and is an innovative commercial mall. The cycling OD data of this area on March 14 are selected to predict the supply and demand of shared bicycles by using O and D models of commercial building AOIs. The predicted bike OD numbers of Fenghuang Square are shown in Figure 8. The result also reflects the characteristics of the morning and evening peaks. The number of bicycle destination points in the morning peak hours was slightly higher than that of origin points, while the number of origin points in the evening peak hours was much higher. This prediction is in line with the regularity of the use of bicycles near commercial buildings.

### Model Evaluation

Bicycles have different cycling patterns in different types of AOIs. Using the constructed prediction model, bicycle supply and demand prediction models suitable for different types of AOIs, including residential areas, commercial buildings, scenic spot areas, science, education and cultural areas, and other service areas, are obtained after model training. The data used in model training are the series data of each type of AOI in the whole study area, with a total of 32 days of data, 80% of which is 25 days in total as the training set, and 20% of which is 7 days in total as the test set.

**Figure 9:**
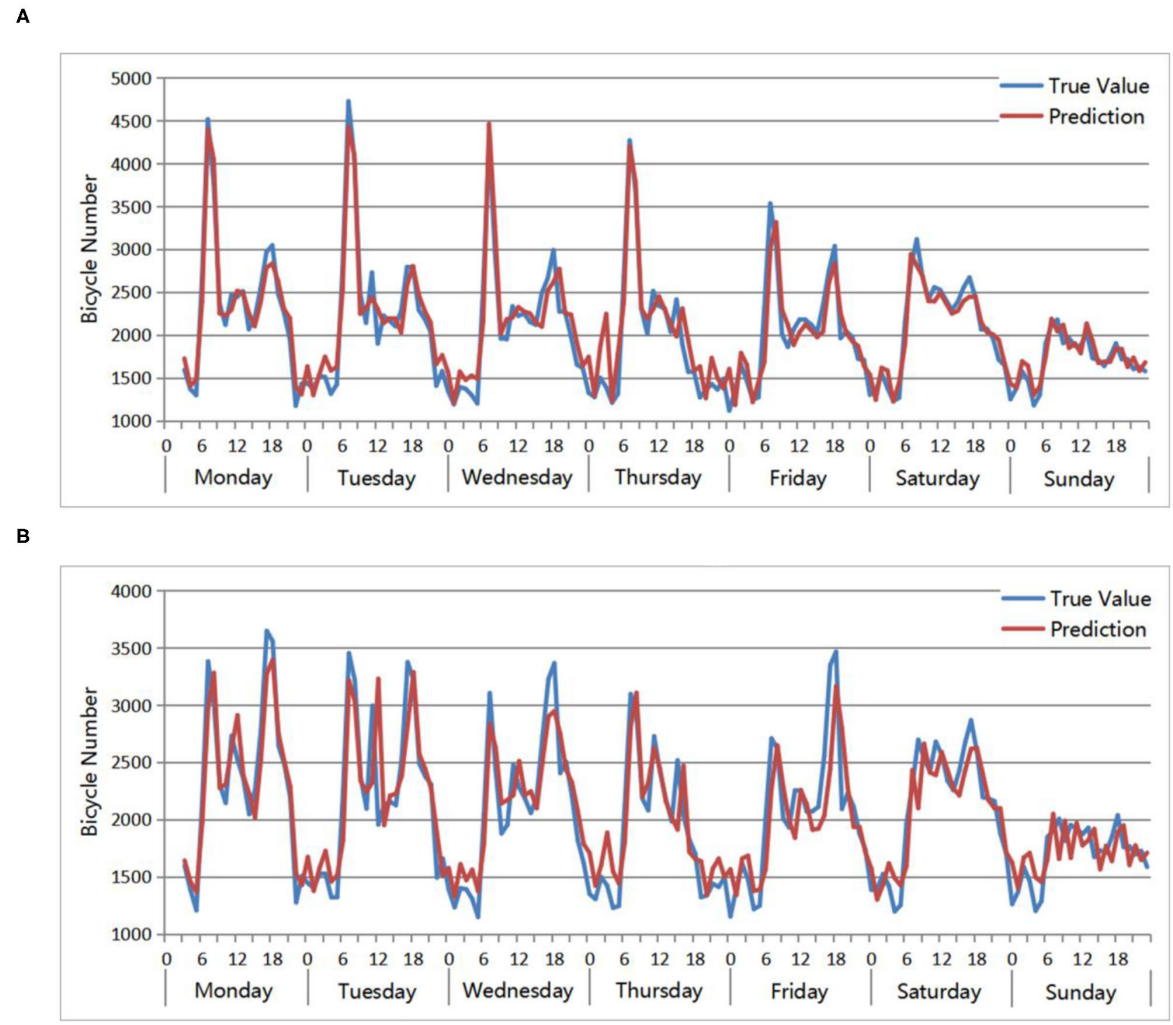
Accuracy evaluation: true value and prediction of QPSO-LSTM in residential areas. **(A)** Origin points number. **(B)** Destination points number.

Figure 9 verifies the prediction accuracy of the OD distribution models of the residential areas. When verifying the model, the data from the week from March 12th to March 18th are selected as input. Each day consists of 24 h of data, with a total of 7^*^24, 168 pieces of input data. Since the time step set in the training model is 4, a total of 168^*^4 inputs are formed after conversion to supervised learning, and the range of predicted results is from 3 o'clock on March 12 to 23 o'clock on March 18. The results show that the predicted value and the true value of the QPSO-LSTM models are relatively consistent, as well as the change trend in the values. The peak times appearing each day are consistent, and the peak values are similar. In addition, residents have strong cycling regularity on weekdays but no obvious cycling regularity on weekends. There are several obvious morning peaks and evening peaks within a week, but the use of bicycles on weekends is greatly reduced. Specifically, for the origin points of the residential areas, as shown in Figure 9A, morning peaks are significantly higher than evening peaks, while for the destination points, as shown in Figure 9B, the comparison results are the opposite. The above conclusions are in line with the cycling characteristics of urban residents and verify the performance of the QPSO-LSTM prediction model.

**Figure 10:**
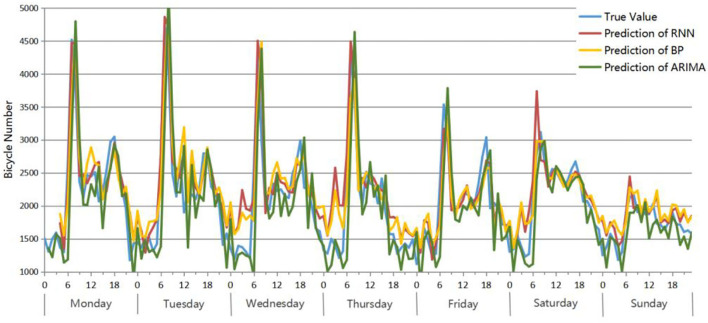
Accuracy evaluation: True value and prediction results of 3 models for the residential area bicycle origin point number from 4:00 on March 12 to 23:00 on March 18.

The BP network, RNN network and ARIMA model are applied to predict the number of bicycle origin points of residential areas from 4:00 on March 12 to 23:00 on March 18. The comparison results between the true value and prediction of these three models are shown in Figure 10. Comparing the prediction results of the three models with the true value, the change trends are roughly the same, and they all perform well at predicting the number of bicycles between peaks. However, there are some differences in the predictions for the periods around the peaks. The predicted values of ARIMA during these periods are closer to the true values than the other two models, especially in the periods near the low peaks. For example, from 0:00 to 6:00 every day, the ARIMA prediction is closer to the true value, while the prediction values of RNN and BP are significantly higher. In addition, compared with the predictions of BP and RNN, BP performs slightly better than RNN. As a result, ARIMA has the best prediction effect, followed by BP and finally RNN.

To verify the accuracy of the QPSO-LSTM model, a verification set is used to compare and verify the prediction accuracy of the other models constructed, including the BP network, RNN network, and ARIMA model, as shown in Table 4. Whether RMSE, MAE, or MAPE is used as the evaluation index, the error of the QPSO-LSTM model is smaller than that of RMSE, MAE, or MAPE, which means that the prediction accuracy of the QPSO-LSTM model is higher than that of theirs. Taking MAPE as an example, the QPSO-LSTM model has the lowest error value, which is 0.087. Conversely, it has the highest accuracy, which is up to ~91%. The error values of the other three models are all >0.1, which means that their accuracy is <90%. As a result, the QPSO-LSTM model is confirmed to have better accuracy and to be able to reasonably predict the supply and demand of shared bicycles of different AOIs.

**Table 4:** Accuracy comparison of different models.

**Type**	**RMSE**	**MAE**	**MAPE**
BP Network	354.58	247.99	0.131
RNN Network	346.47	266.01	0.145
ARIMA Model	314.13	193.12	0.104
QPSO-LSTM Model	224.63	160.40	0.087

## Conclusions

The travel mode is a factor influencing environmental and public health that cannot be ignored (58, 59). Shared bicycles, as a healthy and environmentally sustainable travel mode, should be conveniently accessed by the public. Thus, it is necessary to predict the demand for shared bicycles and optimize the supply of shared bicycles in different urban regions. In this article, a bicycle prediction model called QPSO-LSTM is established, which aims to predict the number of bicycles at OD points in different regions. It is trained with the same dataset as BP, RNN, and ARIMA, and the result shows that QPSO-LSTM significantly outperforms the other models. Furthermore, the model is also applied to predict the bicycle numbers in two typical areas, and the prediction results validate the availability of the model.

Since QPSO-LSTM can predict the future supply and demand for shared bicycles of each analysis unit (AOI), the future distribution of shared bicycles can be rebalanced based on the prediction results. By designing scheduling schemes that optimize resource allocation, idle bikes can be dispatched in a timely manner to areas with large demand, so that the number of shared bicycles tends to be reasonable. Reasonable scheduling optimization can rebalance the distribution of shared bicycles, meet the needs of users for using bikes, improve user satisfaction, and ensure that the shared bicycle system is in a state of dynamic balance. Optimization of bicycle layout according to the actual demand and time series regularity can effectively guide the planning of urban green travel.

Although the QPSO-LSTM has been verified to be a useful method to predict the demand for shared bicycles, some limitations remain.

(1) The current QPSO-LSTM mainly used cycling data, and the weather data (e.g., temperature, wind speed and precipitation) were incorporated into the cycling data. However, other factors such as road networks, visibility in foggy weather, and seasonal changes in the weather might also affect the public's willingness to use bicycles and cause differences in OD distributions. Thus, these factors should also be taken into account in the prediction model.

(2) The performance of QPSO-LSTM was evaluated by using data from Nanjing. However, whether the model is a good choice for other similar cities still needs to be tested. In addition, the characteristics of dockless shared bicycle OD trips need to be analyzed to discover the driving mechanism of these characteristics.

(3) The prediction of the OD distribution provides opportunities for optimizing shared bicycle allocation. However, there is still a long way to go to keep the demand and supply of shared bicycles in a dynamic balance. The optimization of the layout of shared bicycles based on the prediction results is expected to be explored in further studies.

## Data Availability Statement

The data and codes that support the findings of this study are available in GitHub with the identifier: https://github.com/SharingBikeNNU/QPSO-LSTM.

## Author Contributions

MC: conceptualization, investigation, writing, and funding acquisition. YL: methodology and validation. YZ: methodology. GL: project administration. ZM: investigation and writing—review and editing. All authors contributed to the article and approved the submitted version.

## Funding

This study was supported by the National Natural Science Foundation of China (Grant Nos. 41671385, 41622108, and 41871178), the Priority Academic Program Development of Jiangsu Higher Education Institutions (No. 164320H116).

## Conflict of Interest

The authors declare that the research was conducted in the absence of any commercial or financial relationships that could be construed as a potential conflict of interest.

## Publisher's Note

All claims expressed in this article are solely those of the authors and do not necessarily represent those of their affiliated organizations, or those of the publisher, the editors and the reviewers. Any product that may be evaluated in this article, or claim that may be made by its manufacturer, is not guaranteed or endorsed by the publisher.
